# FORTIFYING THE FUTURE OF GERONTOLOGY AND GERIATRICS IN HIGHER ED: PART I—HARD QUESTIONS

**DOI:** 10.1093/geroni/igae098.1034

**Published:** 2024-12-31

**Authors:** Rona Karasik, Julie Masters, Marilyn Gugliucci

**Affiliations:** Chair; Co-Chair; Discussant

## Abstract

Academic programs in gerontology and geriatrics are again facing an uncertain future. Despite the much touted increase in older adults in the US and subsequent demand for an age-savvy workforce, many gerontology and geriatrics programs are struggling to attract enrollments sufficient to survive, much less thrive. At the same time, unprecedented budget woes and declining public support throughout higher education are driving deep cuts across academic programs and disciplines. Yet, for students whose passion is to work in the field of aging as a clinician, researcher, or provider, having programming available is a necessity. This symposium examines the current state of educational programs in gerontology and geriatrics and raises essential questions to consider in order to fortify their future. First, Mark Staley and Julie Masters address the question “What is happening to gerontology programs?” and present an updated census of gerontology programs in the US, highlighting the financial health of academic institutions where programs have closed, paused, or folded into other disciplines. Second, Rona Karasik identifies research and practice trends in Gerontology & Geriatrics Education journal submissions that seek to answer the question “Why don’t students choose to go into gerontology or geriatrics?” Third, Isaac Boateng, Mark Staley, and Jessica VanderWerf share current gerontology students’ perspectives regarding “Why DO students choose gerontology?” Fourth, Julie Masters poses challenging questions that must be considered when devising strategies for a strong and sustainable future for gerontology education. Finally, discussant Marilyn Gugliucci will facilitate a conversation on the state of gerontology and geriatrics education.

